# Comparison of surgical payer costs and implication on the healthcare expenses between laparoscopic magnetic sphincter augmentation (MSA) and laparoscopic Nissen fundoplication (LNF) in a large healthcare system

**DOI:** 10.1007/s00464-019-07021-4

**Published:** 2019-08-02

**Authors:** Shahin Ayazi, Ali H. Zaidi, Ping Zheng, Kristy Chovanec, Nobel Chowdhury, Madison Salvitti, Kirsten Newhams, Jonathan Levy, Toshitaka Hoppo, Blair A. Jobe

**Affiliations:** Esophageal and Lung Institute, Allegheny Health Network, 4815 Liberty Avenue, Suite 439, Pittsburgh, PA 15224 USA

**Keywords:** Gastroesophageal reflux disease (GERD), Nissen fundoplication, Magnetic sphincter augmentation, Cost

## Abstract

**Introduction:**

Magnetic sphincter augmentation (MSA) is a promising antireflux surgical treatment. The cost associated with the device may be perceived as a drawback by payers, which may limit the adoption of this technique. There are limited data regarding the cost of MSA in the management of reflux disease. The aims of the study were to report the clinical outcome and quality of life measures in patients after MSA and to compare the pharmaceutical and procedure payer costs and the disease-related and overall expense of MSA compared to laparoscopic Nissen fundoplication (LNF) from a payer perspective.

**Methods and procedures:**

This prospective observational study was performed in conjunction with the region’s largest health insurance company. Data were collected on patients who underwent MSA over a 2-year period beginning in September 2015 at the study network hospitals. The LNF comparison group was procured from members’ claims data of the payer. Inclusion was predicated by patients having continuous coverage during study period. The total procedural reimbursement and the disease-related and overall medical claims submitted up to 12 months prior to surgery and up to 12 months following surgery were obtained. The payer reimbursement data are presented as allowed cost per member per month (PMPM). These values were then compared between groups.

**Results:**

There were 195 patients who underwent MSA and 1131 that had LNF. MSA results in comparable symptom control, PPI elimination rate, and quality of life measures compared to values reported for LNF in the literature. The median (IQR) reimbursement of surgery was $13,522 (13,195–14,439) for those who underwent MSA and $13,388 (9951–16,261) for patients with LNF, *p* = 0.02. In patients who underwent MSA, the median reimbursement related to the upper gastrointestinal disease was $ 305 PMPM, at 12 months prior to surgery and $ 104 at 12 months after surgery, representing 66% decrease in cost. These values were $ 233 PMPM and $126 PMPM for patients who underwent LNF, representing a 46% decrease (*p* = 0.0001). At 12 months following surgery, the reimbursement for overall medical expenses had decreased by 10.7% in the MSA group and 1.4% in the LNF group when compared to the preoperative baseline reimbursement. The reimbursement for PPI use after surgery showed a 95% decrease in the MSA group and 90% among LNF group when compared to the preoperative baseline (*p* = 0.10).

**Conclusion:**

When compared with LNF, MSA results in a reduction of disease-related expenses for the payer in the year following surgery. While MSA is associated with a higher procedural payer cost compared to LNF, payer costs may offset due to reduction in the expenses after surgery.

Gastroesophageal reflux disease (GERD) is defined as reflux of gastric contents into the esophagus causing troublesome symptoms and complications. This disease is often a chronic condition and affects approximately 25% of the adult population in the USA and is the most common gastrointestinal indication for seeking medical attention worldwide [1, 2]. Gastroesophageal reflux disease also accounts for considerable health care utilization and spending. In 2015, annual health care expenditures for esophageal disease in the United States were estimated to be $18.1 billion, making it the costliest gastrointestinal illness [3].

Acid suppression therapy with proton-pump inhibitors (PPIs) is an effective first-line therapy for controlling the symptom of heartburn and healing erosions in most patients with GERD. However, nearly 40% of patients experience breakthrough symptoms with PPI use [1]. There are also multiple potential risks associated with long-term use of PPIs that have been suggested in cross-sectional cohort studies. Laparoscopic Nissen fundoplication is a safe, effective, and durable treatment. However, patients undergoing a Nissen fundoplication are at risk of potential side effects of the procedure, such as the gas bloat syndrome, the inability to belch and vomit, and the occurrence of persistent dysphagia that may require endoscopic intervention or revisional surgery [4]. The limitations of pharmacologic therapy and fundoplication leave many patients and clinicians in the ambiguous position to either tolerate a life-time of drug dependence with incomplete symptom relief or to undertake the risk of a surgical procedure that may have considerable side effects. The LINX Reflux Management System (Ethicon, Johnson & Johnson, Shoreview, MN) was developed to address the need for an alternative treatment option in the management of patients with GERD, through a safe and reproducible laparoscopic procedure, that does not alter gastric anatomy, augments the anatomic barrier to reflux, and can be reversed if necessary.

Studies have demonstrated that MSA is a safe and effective treatment with symptom control, freedom from PPI and pH normalization rates comparable to those reported for laparoscopic Nissen fundoplication [5–8]. Although studies suggest that the side effect profile of MSA is better than LNF as evidenced by less gas bloating and increased ability to belch and vomit [9], many payers are hesitant to cover the costs of this procedure. The cost associated with the device itself which averages $5000 may be perceived as a drawback that may limit the adoption and use of this technique. There is a paucity of data in regard to the cost analysis of MSA in management of GERD but published data suggest that MSA is associated with shorter operative times and shorter length of stay compared to LNF [10].

Due to the enormous economic burden of GERD, there is great interest in identifying the most cost-effective strategies in management of this disease. Therefore, cost analyses have been performed comparing PPIs versus histamine (H2)-receptor antagonists for acid suppression, various PPIs and dosing strategies, empiric medical treatment versus endoscopy-oriented treatment and PPIs vs. laparoscopic fundoplication. The magnetic sphincter has been FDA approved for more than 6 years and to date no studies have aimed to compare the cost-effectiveness of this device to laparoscopic Nissen fundoplication, the current standard surgical treatment. Therefore, we designed the current study for the following goals: (1) to evaluate the clinical outcome and GERD-Health-Related Quality of Life (GERD-HRQL) in patients having undergone MSA to enable readers to compare them to the well-established historical values reported for LNF and (2) to compare the procedure cost and the overall and disease-related economic impact of MSA compared to laparoscopic Nissen fundoplication (LNF) using claims data.

## Methods and procedures

### Study design

This is a retrospective review of prospectively collected data from a single vertically integrated healthcare system. Clinical data were collected on all patients undergoing MSA between 2015 and 2017, under the approval of the Institutional Review Board at the Allegheny Health Network (AHN). Additionally, Highmark Health shared deidentified aggregated cost data for MSA and LNF groups with approval from the institutional Data Governance Committee. Highmark Health is the region’s largest health insurer that serves approximately 5 million members in Western and Central Pennsylvania, the Lehigh Valley, West Virginia, and the border areas of eastern Ohio.

This work was completed in concert with the VITAL program within Highmark Health. Highmark Health is the second largest integrated health care delivery and financing network in the nation. This program was developed in order to create the optimal environment for the assessment of novel health care technologies and methods with the goal of reducing cost while improving care and mitigating risk.

Inclusion criteria included patients with objective evidence of GERD, who were between the age of 18–80 years and maintained coverage in a Highmark insurance plan up to 12 months prior to surgical intervention for treatment of their reflux disease and during the analysis period.

### Patient populations

#### MSA group

Data were collected on patients who underwent MSA over a 2-year period beginning in September 2015 at the hospitals affiliated to Allegheny Health Network (AHN). The surgery was performed by any one of the four foregut surgeons within the Esophageal and Lung Institute within AHN. The decision to repair hiatal hernia was made intraoperatively by the operating surgeon. In most cases, a posterior mediastinal dissection of the esophagus was performed to the level of the carina and posterior crural closure was performed after restoration of intra-abdominal esophageal length.

All patients were asked to complete validated questionnaires preoperatively and at 6 and 12 months postoperatively. The validated questionnaires included GERD-HRQL and Reflux Symptom Index (RSI). The GERD-HRQL consists of 10 questions which specifically address GERD symptoms [11]. Each question has a score ranging from 0 to 5, and the best possible aggregate score is 0 (asymptomatic), and the worst score is 50 (very severe symptoms). A total score of ≥ 10 is considered abnormal. The RSI was used to assess atypical GERD symptoms [12]. The RSI consists of 9 questions, and each question has a potential score ranging from 0 to 5. A total score > 13 is considered abnormal. Total raw scores were calculated by summing the score for each item to yield a score between 10 and 31 and were recalibrated to interval-level scores from 0 to 100, with higher values indicating greater disease severity. Comparing the postoperative scores with the preoperative scores on each questionnaire, the rate of symptomatic improvement was calculated.

#### LNF group

This comparison group was procured from members’ claims data of the Highmark Health and included patients who have been treated either at AHN hospitals or non-AHN hospital within Western Pennsylvania but covered by payer in this study. Clinical and outcome data were not available for this group of patients.

### Cost analysis

To analyze health care utilization and cost of care, Highmark claims data were used for both MSA and LNF groups to capture the per member per month (PMPM). This value represents the average amount per month that subscribing members cost their insurance providers. The total procedural cost and the disease-related and overall claims and claims limited to PPIs use submitted up to 12 months prior to surgery and 12 months following surgery were obtained from insurance company and reported as aggregate PMPM.

For disease-related costs, relevant ICD-10 and CPT codes were utilized. All costs were reported as weighted averages for 6-month and 12-month coverage windows.

Additionally, for inclusion into the analysis each individual subject was required to have equal number of months of claims data pre- and post-procedure. Comparative LNF data were assembled under the same guidelines from eligible patients for the same time window. These values were then compared between groups.

### Statistical analysis

Values are expressed as either mean with standard deviation (SD) or median with interquartile range (IQR) when appropriate. Statistical analysis was performed by means of non-parametric Mann–Whitney *U* test, Wilcoxon signed-rank test, and Person’s *χ*^2^ test when appropriate. A *p* value < 0.05 was considered significant. Statistical analysis was performed using SAS software (SAS Institute Inc., Cary, N.C.).

## Results

A total of 1311 patients met the inclusion criteria for this study. There were 195 patients in the MSA and 1131 in the LNF groups. In the MSA group, 180 patients maintained their insurance coverage during analysis window and were included in the cost analysis. Baseline demographic and clinical data for the MSA group is shown in Table 1.

**Table 1:** MSA patients’ characteristics at baseline

Characteristics	*N* (%)
Age (year)	
Mean (SD)	52.7 (14.1)
Gender	
Male	78 (40.0)
Female	117 (60.0)
BMI	
Mean (SD)	29.1 (4.6)
Hiatal hernia	
Yes	164 (84.1)
No	31 (15.9)
Size of hiatal hernia	
Small (≤ 3 cm)	116 (70.7)
Large (> 3 cm)	39 (23.8)
PEH	9 (5.5)

### Outcome and quality of life in MSA

**Fig. 1:**
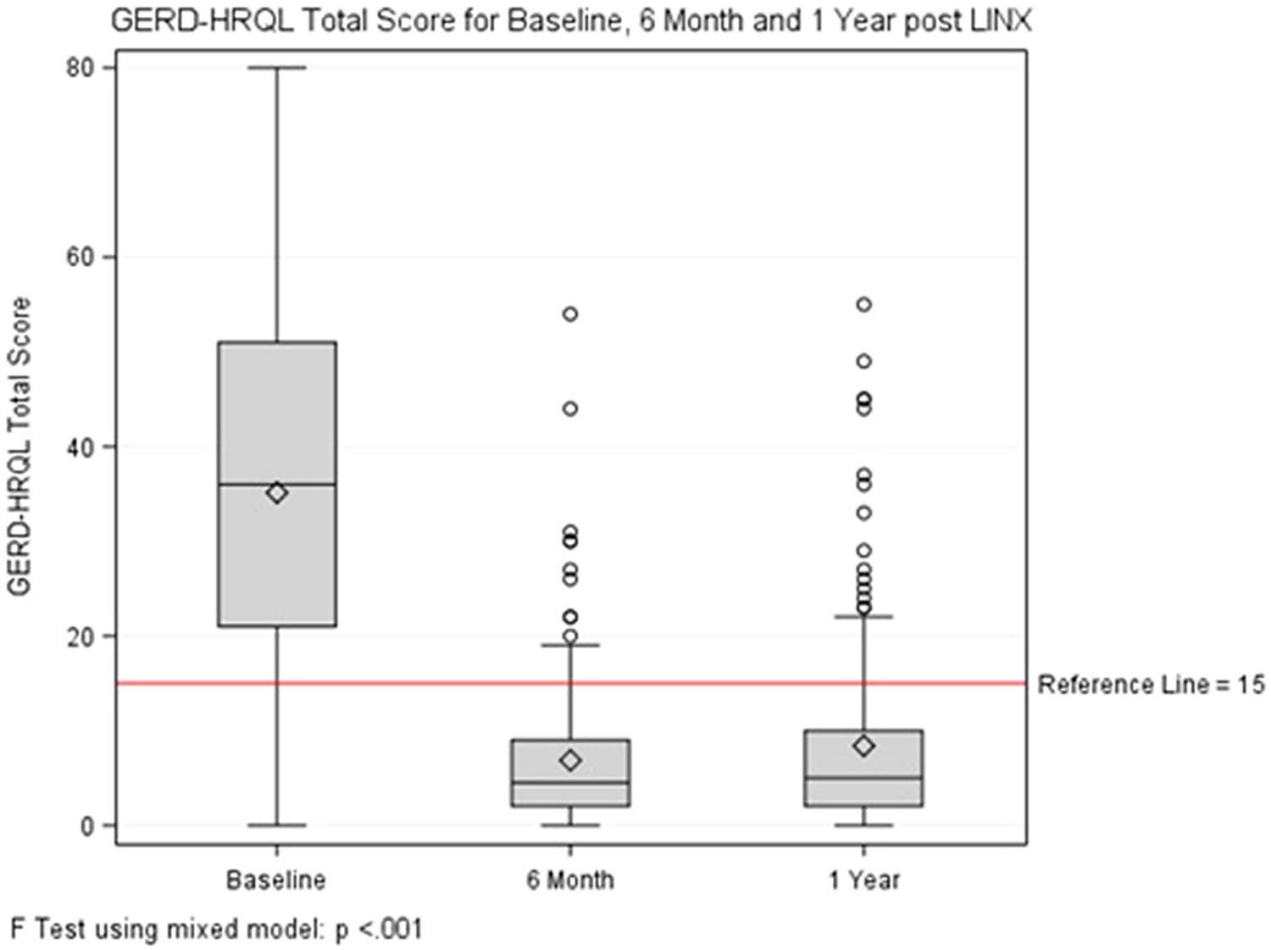
GERD-HRQL total score for baseline, 6 months and 1 year after MSA

**Fig. 2:**
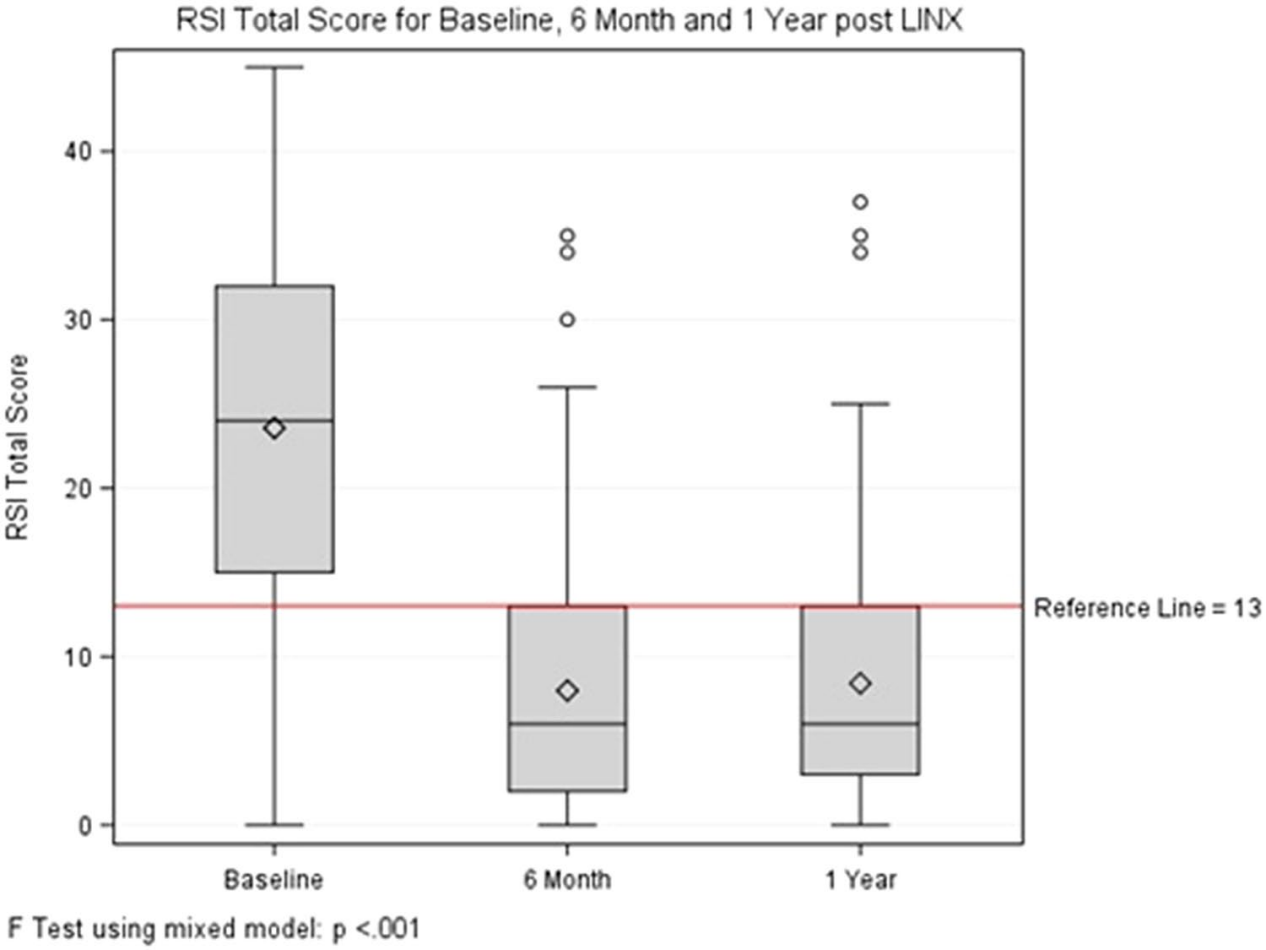
RSI total score for baseline, 6 months and 1 year after MSA

At a mean follow-up of 13.8 months, 5 (2.6%) patients required removal of the magnetic ring primarily for persistent dysphagia. Most patients were discharged home on the day of surgery (89%) and only 16 required overnight stay at a mean of 1.6 days. At 1-year follow-up, 90.7% of patients were satisfied with the outcome of their surgery and 91.8% were off PPIs. Comparison of QOL measures for baseline values and 12 months after surgery demonstrated normalization of scores in the majority of cases (Table 2). GERD-HRQL total score was 34.8 (18.6) at baseline and decreased to 6.9 (7.9) at 6 months and 8.2 (9.6) at 12 months (Fig. 1, *p* < 0.001 for both comparisons). Similar comparison for RSI total score is shown in Fig. 2.

**Table 2:** Comparison of QOL measures for baseline and 1-year post-LINX implantations

Measurement	Mean (SD)		*p* value
	Baseline	1 year	
GERD-HRQL scoring			
Heartburn score	15.1 (8.6)	2.8 (4.6)	< 0.001
Base score	21.3 (11.5)	6.0 (6.3)	< 0.001
Regurgitation score	13.8 (9.1)	2.2 (4.2)	< 0.001
Total score	35.1 (19.2)	8.2 (9.6)	< 0.001
Satisfaction	0.6%	90.7%	< 0.001
Clinical improvement	133 (80.1%)		N/A
PPI use	86.7%	8.2%	< 0.001
RSI scoring			
Difficulty swallowing score	1.7 (1.6)	1.0 (1.2)	< 0.001
Difficulty swallowing score ≥ 3	35.0%	11.7%	< 0.001
Total score	23.5 (10.6)	8.4 (7.5)	< 0.001
Total score ≥ 13	81.0%	25.2%	< 0.001

### Surgical cost and implication on the healthcare expense

Based on payer data, the median (IQR) same-day payer cost of surgery was $13,522 (13,195–14,439) for those who underwent MSA and $13,388 (9951–16,261) for patients with LNF, *p* = 0.02. In patients who underwent MSA, the median reimbursement related to the upper gastrointestinal disease was $305 PMPM, at 12 months prior to surgery and $104 at 12 months after surgery, representing 66% decrease in cost. These values were $233 PMPM and $126 PMPM for patients who underwent LNF, representing a 46% decrease (Table 3).

**Table 3:** Comparison of overall and disease-related payer costs (PMPM), 12 months before and 12 months after surgery

	12 months prior to surgery	12 months after surgery	% reduction in cost	*p* value*
Overall medical reimbursement				
MSA	$1115	$996	10.7	> 0.05
LNF	$1272	$1254	1.4	
Disease-related medical reimbursement				
MSA	$305	$104	65.9	0.0001
LNF	$233	$126	46.0	

At 12 months following surgery, the reimbursement for overall medical expenses had decreased by 10.7% in the MSA group and only 1.4% in the LNF group when compared to the preoperative baseline reimbursement data (Table 3). These analyses were repeated by comparison of data 6 months prior to surgery to 6 months after surgery and the decrease in disease-related reimbursement was again significantly different (Table 4).

**Table 4:** Comparison of overall and disease-related payer costs (PMPM), 6 months before and 6 months after surgery

	6 months prior to surgery	6 months after surgery	% reduction in cost	*p* value^a^
Overall medical reimbursement				
MSA	$1216	$1039	14.5	> 0.05
LNF	$1544	$1381	10.6	
Disease-related medical reimbursement				
MSA	$443	$86	80.7	0.0001
LNF	$337	$127	62.1	

^a^For comparison between reduction in costs

**Fig. 3:**
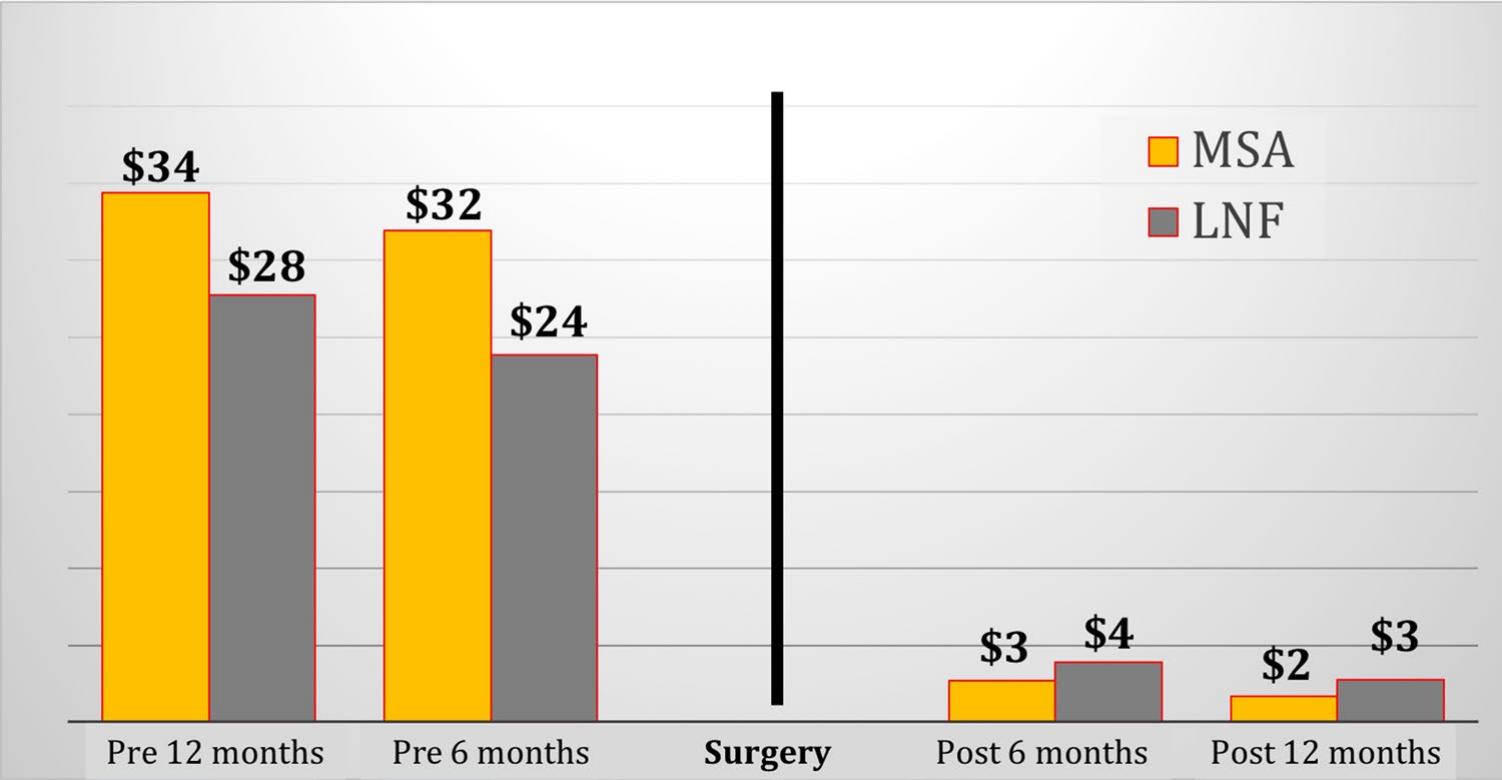
Comparison of PPI payer costs (expressed as PMPM) between MSA and LNF groups before and after surgery

The reimbursement for PPI use after surgery showed a 95% decrease in the MSA group and 90% among LNF group when compared to the preoperative baseline (*p* = 0.10), (Fig. 3).

## Discussion

Gastroesophageal reflux disease (GERD) is a chronic condition and frequently requires prolonged or definitive therapy. Patients with a diagnosis of GERD have a higher incidence of a subsequent diagnosis of esophageal adenocarcinoma, esophageal stricture, chronic cough, sinusitis, asthma, and sleep problems [13]. GERD is also an exceedingly common disease, ranking as the most frequent gastrointestinal diagnosis associated with outpatient clinic visits in the United States, with nearly 9 million visits in 2009 [14]. Previous studies have demonstrated that GERD affects between 18% and 27% of individuals in the United States [15]. This disease also has a profound effect on quality of life and work performance. All these factors make GERD an expensive disease to manage.

The direct costs associated with GERD in the USA were estimated to be $12.1 billion in 2004, making it the costliest gastrointestinal illness. Proton-pump inhibitors (PPIs) account for the majority of GERD-related spending, and an estimated $9.4 billion per year is spent on PPIs in the USA alone [3]. In fact, among the list of the ten costliest prescription medications for alimentary tract illnesses, the top five are various forms of PPIs [16]. PPIs also account for two of the top-five selling drugs in the United States [2].

The treatment options for patients with chronic gastroesophageal reflux disease have been dominated by two primary options: Nissen fundoplication and antisecretory medication therapy, first with histamine receptor antagonists and later with PPIs. The common first-line treatment for GERD is the use of PPIs, which have been demonstrated to be effective for both healing of erosions and control of heartburn. However, nearly 40% of patients experience breakthrough symptoms [17, 18]. Moreover, evidence is emerging that long-term use of PPIs may be associated with infectious complications, cardiac sequelae, nutritional deficits, and even dementia [19].

The Nissen fundoplication, introduced more than half a century ago, still represents the surgical standard of care and a treatment option usually reserved for patients whose condition has failed to respond to medical therapy or who desire to be free from dependence on medical therapy. The current approach to laparoscopic Nissen operation is a safe, effective, and durable therapy [20]. This operation is, however, underused due to the fear of long-term side effects and failure, which impact referral patterns [21]. Currently, fewer than 30,000 Nissen fundoplication procedures are performed annually in the USA, corresponding to less than 1% of the GERD population [22].

The LINX Reflux Management System (Ethicon, Johnson & Johnson, Shoreview, MN) was developed to address the need for alternative treatment options in management of patients with GERD. This device was approved by FDA in 2012 and several studies have confirmed its efficacy, safety, and durability [10, 23]. This procedure is performed laparoscopically and does not alter gastric anatomy. It applies magnetic force to augment the barrier function of the LES and can be reversed if necessary. Despite its efficacy and low side effect profile, insurance carriers have been hesitant to cover MSA as a surgical option for reflux disease. This is mainly due to their concern for additional costs of the procedure and stems from lack of cost analysis in regard to use of MSA in management of patients with GERD.

Rising healthcare costs pose significant concerns to system viability; thus, improving outcomes while restricting costs is a primary concern of reform efforts around the world. A better understanding of the clinical and economic implications that may be associated with surgical therapy for GERD will help health system decision-makers make the proper trade-offs in a period of financial constraints and when there is a need for optimal resource allocation.

In an increasingly cost-conscious healthcare environment, clinicians must remain mindful of the costs associated with any treatment modality by current evidence-based guidelines. The magnetic sphincter augmentation is a promising new innovation in the surgical management of reflux disease. The best innovation increases therapeutic efficacy but costs less than standard therapy. While this is a laudable goal, most innovations increase both cost and effectiveness and the trade-off between the increased cost and effectiveness must be determined by studies evaluating economic impact of a treatment option in management of a disease. To address this need, the current study was performed to evaluate and compare the clinical and economic consequences associated with MSA and LNF 1 year before and 1 year after the surgical procedure.

The major finding of our study is that implementation of both types of surgery decreased overall and disease-related costs over 6- and 12-months after the procedure. When compared with LNF, however, MSA results in a higher reduction of disease-related expense in the year following surgery. While MSA is found to be associated with a higher same-day surgical cost compared to LNF, mainly driven by the cost of device itself, the higher cost can partially offset by reduction in hospital cost due to shorter hospital stay. The patients are routinely discharged on the same day after MSA. Only 16 patients (11%) required overnight stay in MSA group and mean hospital stay for these 16 patients was 1.6 days. The only other publication that has studied charges related to MSA reported that the additional charge for the MSA device itself was completely offset by shorter operative times and length of stay (LOS) for MSA. In this study, Reynolds and colleagues [9] showed that the shorter LOS by about a day resulted in fewer laboratory tests, less medication usage of narcotics and antiemetics, and decreased charges for room and board as most patients were discharged from the recovery room, whereas LNF patients were admitted and stayed one night. This resulted in overall charges for MSA being equal to LNF in their study.

We found a significant variability in the procedure costs among LNF group, evidenced by a wide range of IQR ($ 9951–16,261) and an SD that was more than twice of the MSA group ($7950 vs. $3472). This variation in the costs is the result of variation in the delivery of Nissen fundoplication among different centers. Conversely, there was a much narrower IQR among patients with MSA ($13,195–14,439). MSA utilizes a device and this results in a standardized and reproducible procedure and further leads to consistency in the outcome of this procedure among reports from different centers. MSA is shown to be a reproducible surgical procedure for insertion. Since the first implants, consistent performance and reproducibility have been observed in multiple centers worldwide.

We found that MSA resulted in a significant reduction in disease-related reimbursement in the year following surgery compared to LNF. Payer costs may be offset secondary to this reduction in the year following surgery. There was also a trend toward higher reduction in the cost associated with PPI use in the year following surgery in patients with MSA, although the difference did not meet the statistical significance (*p* = 0.10).

The current study calculated the total per member per month (PMPM) reimbursement payment for all of the cost categories and for disease-related claims. PMPM medical reimbursement payments are important to payers because they correspond to the mean expenditures by a person in a month and are defined as the sum of all the medical claims incurred in a given month. These aggregated reimbursement payments for medical services can be used as a proxy for overall disease and cost burden of a particular type of patient over a fixed period of time. They are particularly effective for noting trends in reimbursement payments and understanding differences in and comparing reimbursement payments over time among patients receiving different technologies or treatment interventions.

This is the first study that uses claims data in patients with antireflux surgery. While using these data provides a window to the economic impact of the antireflux surgery in the real world, it also imposes several limitations. First, access to the clinical information of the LNF group was not available. The cost data for this group were directly provided by insurance provider without having access to the baseline demographic or clinical data of this group. This limited the ability to compare the clinical characteristics between the groups. Second, outcome data and quality of life data were also only available for MSA group, and therefore clinical outcome and GERD-HRQL analysis are only reported for patients who underwent MSA. Reviewing these data will enable readers to compare historical values for LNF with our MSA outcomes and provide support that the observed reduction in disease-related expense in the year following surgery in the MSA group does not come at the expense of a worse clinical outcome. Our study does not cover the over the counter PPI costs, as the cost data were provided by the insurance provider with no access to the OTC expenses. However, the majority of patients obtain their PPIs through insurance and the potential bias due to not including the OTC costs will likely affect both groups equally and therefore not impact the overall conclusion of this study. We were also unable to determine the etiology of differences in costs since payer data do not provide the details required to understand cost differential.

Another limitation of this study is that hospital costs were not analyzed or compared as part of this study since it was beyond the scope of the analysis. This study reflects the payer costs based on payer reimbursement for the procedure. Finally, the long-term costs associated with MSA or NF were not considered in the analysis. A recent study showed a 0.3% risk of erosion of the LINX device at 4 years after implantation, which would be associated with costs that were not considered in this analysis [24]. Similarly, explantation of the LINX device or a redo of the NF would incur costs to the payer that have not been considered in this analysis.

In conclusion, patients with GERD who were treated with MSA in a large health network have comparable symptom control, PPI elimination rate, and GERD-HRQL measures compared to values reported for LNF in the literature. In this study, while MSA was associated with a higher same-day surgical costs compared to LNF, disease-related costs to the payers over 12 months post-surgery were lower for MSA compared to LNF.
